# Twitter Poll as a Medium for Questionnaire-Based Health Survey: An Experience of a Pilot Study on the Preference of Systems of Medicine for Various Health Conditions

**DOI:** 10.7759/cureus.28767

**Published:** 2022-09-04

**Authors:** Shaikat Mondal, Purab K Modak, Mohammad Selim, Himel Mondal, Chayan Baidya, Mojca Hribersek, Rajeev K Singla, Bairong Shen, Atanas G Atanasov

**Affiliations:** 1 Physiology, Raiganj Government Medical College And Hospital, Raiganj, IND; 2 Physiology, Sheikh Bhikhari Medical College and Hospital, Hazaribagh, IND; 3 Physiology, Jalpaiguri Government Medical College, Jalpaiguri, IND; 4 Physiology, Saheed Laxman Nayak Medical College and Hospital, Koraput, IND; 5 Panchakarma, Institute of Post Graduate Ayurvedic Education & Research at Shayamadas Vaidya Shastra Pith, Kolkata, IND; 6 Ludwig Boltzmann Institute for Digital Health and Patient Safety, Medical University of Vienna, Vienna, AUT; 7 Institute for Systems Genetics, West China Hospital, Sichuan University, Chengdu, CHN; 8 School of Pharmaceutical Sciences, Lovely Professional University, Phagwara, IND

**Keywords:** questionnaire, health survey, poll, survey, patient opinion, primary care education, twitter, social media platform, survey methodology, public opinion

## Abstract

Background

The easy accessibility of smartphones and internet connections enables people to stay virtually connected to communities via social media. However, social media is also being explored for health care education and dissemination of health-related information. Twitter (Twitter, Inc., San Francisco, California) is one of the popular social media used for spreading health-related information. Twitter enables users to create polls to get opinions from their users. The Twitter poll is a less-explored avenue for health surveys.

Objective

In this pilot study, we aimed to explore the feasibility of conducting a questionnaire-based health survey (on the preference of different systems of medicine for the treatment of various health problems) as a Twitter poll.

Methods

This observational study was conducted on Twitter for five consecutive days starting from May 31, 2021. We posted five Twitter polls, one poll each day, for five days in a #INPST unique Twitter campaign. Preferences on the use of modern medicine, traditional medicine, a combination of these two systems, and self-medication were collected on five health conditions. We collected the data from the landing poll page and Tweet Analytics (insight about the engagement of tweets provided free by Twitter). The Chi-square test, binomial test, and one-way Analysis of Variance were used to compare data, and Spearman's rank correlation coefficient was used to find a correlation between categorical variables.

Results

We had a mean 4358.6±590.3 poll reach with the engagement of 108.2±36.87 Twitter users and 67.6±28.06 votes. Most of the responses were received on the first day of posting the poll. The participation then gradually decreased. Modern medicine was the first choice for emergency medical care (85.1%, P <0.0001), treatment of cancer (43.6%, P <0.0001), and sexual disorder or transmitted diseases (48.9%, P <0.0001). Traditional medicine was the first choice (37.5%, P = 0.63) for the treatment of common illnesses, and a combination of modern and traditional medicine was the first choice (37.5%, P = 0.01) for the treatment of chronic diseases.

Conclusion

A medical survey with short questions with a maximum of four response options can be conducted on Twitter. Survey results can be obtained without any third-party analytic service. The response rate is highest on the first day and participation may decrease when multiple polls are posted within a Twitter campaign. Preference for systems of medicine found in this study can be used for designing large-scale surveys in the future.

## Introduction

Social media has become a new avenue for health research. Along with other social media, the micro-blogging site, Twitter (Twitter, Inc., San Francisco, California), is being used for public health-related studies [1,2]. Twitter has a huge pool of active users. The major limitation of the site is the character restriction (maximum 280 characters in a post). In addition, for a poll, we can set only a maximum of four response options. Several studies have analyzed the content of tweets for health-related issues [3]. It is also a popular method of communication and dispersion of conference and symposium knowledge [4,5]. Moreover, it has been tried in teaching medical students and residents [6]. However, the Twitter poll has rarely been explored as a tool for a health-related survey.

A previous study by Vidal-Alaball et al. used two Twitter polls to get public opinion on telemedicine [7]. Eibensteiner et al. also used two polls to observe people’s willingness to vaccinate against coronavirus disease 2019 (COVID-19) [8]. Multiple polls are being used in connection with meetings, symposiums, and biomedical conferences. It has even been used for students’ assessment and engagement in courses [9]. In this context, multiple poll-based surveys such as Twitter polls would be a new way of conducting social media surveys. Hence, we planned to conduct a pilot survey with multiple questions posted as a Twitter poll.

Modern medicine and traditional medicine both have been in practice worldwide. People have the cafeteria choice to select any of the medicine systems for their treatment [10]. However, specific preference of a particular system in various health situations (e.g., common illness, cancer, chronic diseases) is not available from social-media research. Hence, we selected this topic for the pilot survey. Primary care physicians would get a glimpse of how people may seek help for different types of illnesses.

A Twitter hashtag networking campaign (#INPST) was held by International Natural Product Sciences Taskforce, an international collaboration platform [11]. We planned to conduct a five-question survey on Twitter along with the #INPST campaign. This study aimed to observe the feasibility of conducting a short health survey with the help of a Twitter poll and to observe the pattern of participation in the poll. Furthermore, the poll result on the preference for systems of medicine for various health conditions would be used to frame one large-scale social media-based health survey in the future.

## Materials and methods

Ethics

Ethical approval was not required for this study. A Twitter poll is an anonymous data collection method. Twitter does not divulge who had participated in the poll. The poll runs in the public domain and anyone, with a free account, can access the result. However, if any users comment, like, or retweet voluntarily for showing their engagement, their profile can be viewed. To maintain the utmost privacy of the users, we did not use any direct quotes from the replies in the tweet [12,13].

Study type and variables

This was a cross-sectional observational study conducted on social media hosted on the World Wide Web. The study variables were twitter poll impression (number of users who saw the tweet), engagement (number of users who interacted with the tweet), votes (number of users who voted on the poll), like (number of users who hit the like button), retweet (number of users who tweeted the poll), reply (number of users who commented on the poll), and details expand (number of users who clicked on the tweet to view more).

Settings

We created five Twitter polls from the Twitter handle of the first author who had 143 followers. The Twitter polls were posted once a day at approximately 10 am (IST) for five consecutive days starting from May 31, 2021. For posting the poll, we used a personal computer (ASUS VivoBook Max X541N, ASUSTeK Computer Inc., Taipei, Taiwan) connected to the internet and an internet browser (Firefox 88.0.1, Mozilla Corporation, Mountain View, California, United States).

Twitter poll

**Figure 1:**
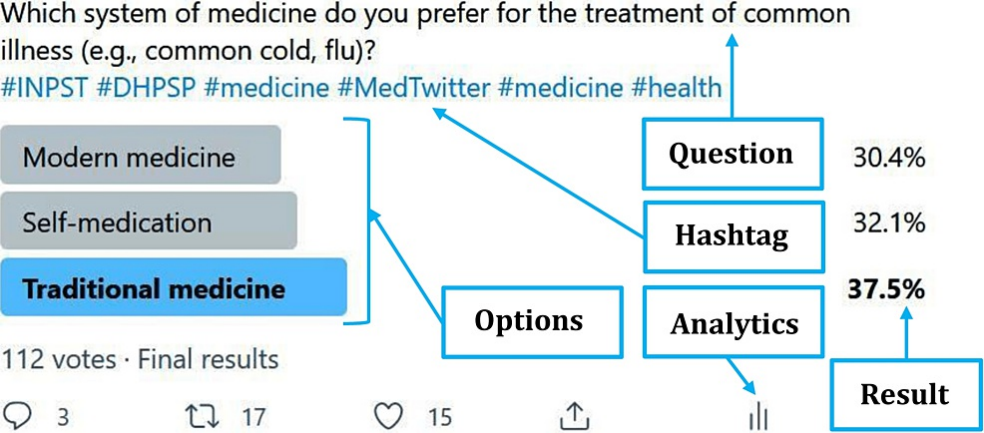
An example of a Twitter poll The screenshot was taken from a Twitter (Twitter, Inc., San Francisco, California) poll used in the study

We posted a poll question with hashtags to target the audience. In this poll, we used three response options. Figure 1 shows a screenshot of our poll. We kept the poll live for seven days. After the specified period, the poll does not take any further responses. The final result of the poll was displayed in percentage on the right side of each option. For analytical purposes, a researcher can convert the percentage to the number of participants who voted for a particular option. In the example, 34 ((112 × 30.4)/100) participants voted for "modern medicine". The number of replies (three in the example in Figure 1), retweets (17 in the example), and likes (15 in the example) can be found below the options. The free "Tweet Analytics" would take the user to further insight about the total impression (how many people have been reached), total engagement to the tweet, details expanded by users, etc. These data can be used for statistical analysis.

Pilot survey poll

There were five questions with variable response options. We posted the poll with relevant hashtags with #INPST and #DHPSP (Digital Health and Patient Safety Platform) as the common hashtags [14]. These questions were reviewed by an expert panel of three persons for content validity. Then, the questionnaire was distributed to five colleagues for a quick pre-testing. All the questions with response options and pre-defined hashtags were kept ready for the week of the #INPST campaign [11]. The poll was open to all. However, each poll was kept online for seven days. After that, the poll is visible to everyone, but not open for voting. This was done as Twitter allows a maximum of seven days to keep the poll online. The poll content is shown in Table 1.

**Table 1:** Twitter poll questions and response options

Poll number	Questions	Response options
Poll 1	Which system of medicine do you prefer for the treatment of common illness (e.g., common cold, flu)?	Modern medicine; Self-medication; Traditional medicine
Poll 2	Which system do you prefer for the treatment of cancer?	Modern medicine; Modern + traditional combo; Self-medication; Traditional medicine
Poll 3	Which system do you prefer for the treatment of chronic diseases (e.g., hypertension, diabetes, asthma)?	Modern medicine; Modern + traditional combo; Self-medication; Traditional medicine
Poll 4	Which system do you prefer for emergency care (e.g., heart attack, road traffic accidents)?	Modern medicine; Traditional medicine
Poll 5	Which system do you prefer for the treatment of sexual dysfunction or sexually transmitted diseases?	Modern medicine; Modern + traditional combo; Self-medication; Traditional medicine

Data collection

The poll result was obtained from Twitter as a percentage. Total impressions, engagements, profile clicks, etc. were collected from Tweet Analytics. All these services are free and provided by Twitter. We did not use any third-party software analytics for the analysis. Only Twitter-provided data were used to get the poll results and patterns of participation. Data were collected daily after posting the first poll. However, the final poll result was considered when the poll is locked by Twitter (i.e., after seven days).

Statistical analysis

Data were presented as numbers, percentages, mean and standard deviations. Descriptive analysis was done to observe the pattern of participation over time. Chi-square test, binomial test, and one-way analysis of variance were used to compare data, and Spearman's rank correlation coefficient was used to find a correlation between categorical variables. For all the statistical tests, we fixed a P <0.05 to be statistically significant. Statistical analysis was done in Microsoft Excel® 2010 (Microsoft Corporation, Redmond, Washington, United States) data analysis tool and GraphPad Prism 6.01 (GraphPad Software, San Diego, California, United States).

## Results

Poll participation

The five polls reached a total of 21,793 (mean 4358.6±590.3) Twitter users with a total of 541 (mean 108.2±36.87) engagements and 338 (mean 67.6±28.06) votes. Poll-wise data is presented in Table 2.

**Table 2:** Impression and participation in the poll *Statistically significant p-value of Chi-square test of data across the column The data indicate the final value got at the end of the seven days

Poll number	Impressions	Total engagement	Votes	Likes	Retweet	Reply	Details expand
Poll 1	5366	159	112	15	17	3	39
Poll 2	4400	136	78	12	16	3	36
Poll 3	3949	86	56	14	14	1	13
Poll 4	3988	80	47	13	11	3	14
Poll 5	4090	80	45	8	7	4	23
Chi-square, p-value	319.8, <0.0001*	50.25, <0.0001*	46.59, <0.0001*	2.36, 0.67	5.08, 0.28	1.7, 0.79	23.44, 0.0001*

There was a gradual decrease in the total engagement and poll participation, with the first posted poll having gained the highest visibility. We found a significant positive correlation of number of votes with impression (r_s_ = 0.96, P = 0.005), engagement (r_s_ = 0.97, P = 0.003), retweet (r_s_ = 0.82, P = 0.044), and details expand (r_s_ = 0.84, P = 0.04). Tweet"‘like" had a non-significant positive correlation (r_s_ = 0.59, P = 0.15) and "reply" had no correlation (r_s_ = 0.005, P = 0.5). The day-wise data is presented in Table 3. The poll reached the highest number of users on the first day of posting. Similarly, the total engagement, votes, and retweets also showed that the first day is the important day for a Twitter poll in a campaign.

**Table 3:** Day-wise data for five polls *Statistically significant p-value of one-way analysis of variance of data across row Day 1 is the first day of a poll and Day 7 is the last day of a poll. Values of five polls of Day 1 were taken to find the mean and standard deviation (SD) and so on.

Variables	Day 1	Day 2	Day 3	Day 4	Day 5	Day 6	Day 7	p-value
	Mean±SD							
Impression	4166±583.99	112.2±26.44	27±10.42	20.2±11.88	13.8±8.79	11.8±7.05	7.6±5.9	<0.0001*
Engagement	96.8±31.06	5.4±6.19	1.2±0.45	4.8±8.58	0.6±1.34	0.8±1.3	0.8±1.79	<0.0001*
Vote	64.6±25.32	2.2±2.86	0.2±0.45	0.4±0.55	0	0	0.2±0.45	<0.0001*
Like	11.2±1.92	0.2±0.45	0	0.2±0.45	0.2±0.45	0.4±0.55	0.2±0.45	<0.0001*
Retweet	11.8±3.7	0.4±0.89	0	0.2±0.45	0.2±0.45	0.4±0.55	0.4±0.55	<0.0001*

Poll results

**Figure 2:**
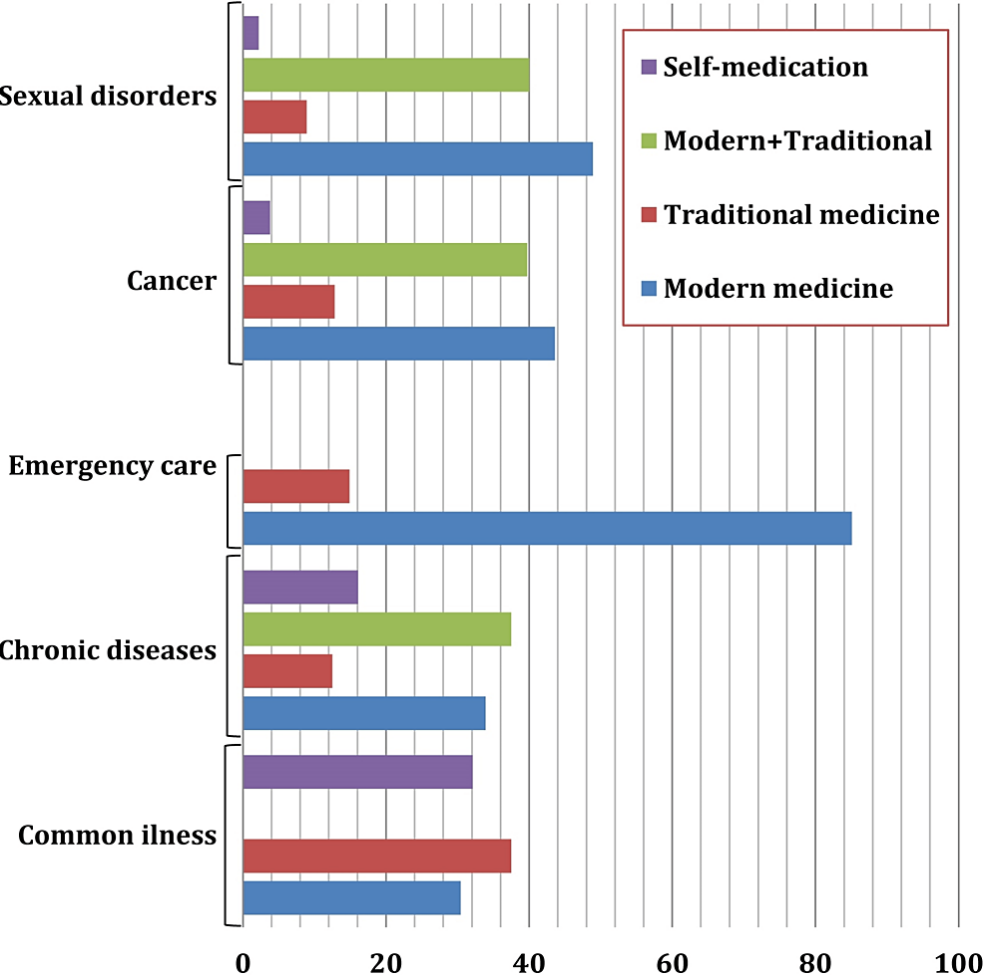
Preference for systems of medicine for various health conditions Minor illness had three options and emergency care had two options. Statistical analysis: Common illness: χ2 = 0.93, P = 0.63; Chronic diseases: χ2 = 10.57, P = 0.01; Emergency care: Binomial test P <0.0001; Cancer: χ2 = 36.15, P <0.0001; Sexual disorder: χ2 = 28.33, P <0.0001

The preference for using different systems of medicine for various health conditions is shown in Figure 2. For common illness, traditional medicine was the first choice (37.5%) followed by self-medication (32.1%) and modern medicine (30.4%) (χ2 = 0.93, P = 0.63). For chronic diseases, a combination of modern medicine and traditional medicine was the first choice (37.5%) and traditional medicine is least preferred as a single method of treatment (12.5%) (χ2 = 10.57, P = 0.01). For emergency medical care, modern medicine is the first choice (85.1%) (binominal test P <0.0001). For the treatment of cancer, although the first choice was modern medicine (43.6%), the combination of modern and traditional medicine was also popular (39.7%) (χ2 = 36.15, P <0.0001). A similar result is found for sexual dysfunction or sexually transmitted infections. The first preference is modern medicine (48.9%). However, combination therapy is preferred by 40% of the users (χ2 = 28.33, P <0.0001).

## Discussion

Feasibility

We surveyed on a social media platform with five questions as Twitter polls. Hence, a series of questions from a questionnaire of a health-related survey can be posted as polls on Twitter. However, a careful design of the survey questions is a prerequisite as the poll needs to be created with just up to 280 characters (inclusive of spaces). The response options also have a restriction of up to 25 characters in each poll choice with a maximum of four choices. A user can set the "Poll length" with an option for selecting days, hours, and minutes. The maximum poll length can be of seven days. This is especially important for time-bound surveys. The user can also select who can reply to the poll: everyone, people you follow, and people you mention. This feature is for close group surveys without interference from the unsolicited response. We conducted our poll within seven days poll length selecting the option that everyone can participate in the poll. 

Response pattern

During the particular hashtag campaign, the vote at our polls showed a gradual decrement. Eibensteiner et al. used two polls in the same thread from the Twitter handle of an organization and got an almost equal number of votes (3439 and 3457) for the polls [8]. Similarly, Vidal-Alaball et al. posted two polls from a user’s handle with 9300 followers with a gap of five months and received 108 and 113 votes respectively [7]. The reason for the gradual decrease in participation in our poll may be multifactorial. The users, who were getting the poll tweet consecutively, might lose interest to participate. Also, the poll questions might not be interesting to the users. However, further research is required to see if such a pattern is seen in any other studies. In the poll, we did not use any reinforcement or announce any incentives for continuous participation in the polls. This may be another reason why participation was not constant [15].

**Figure 3:**
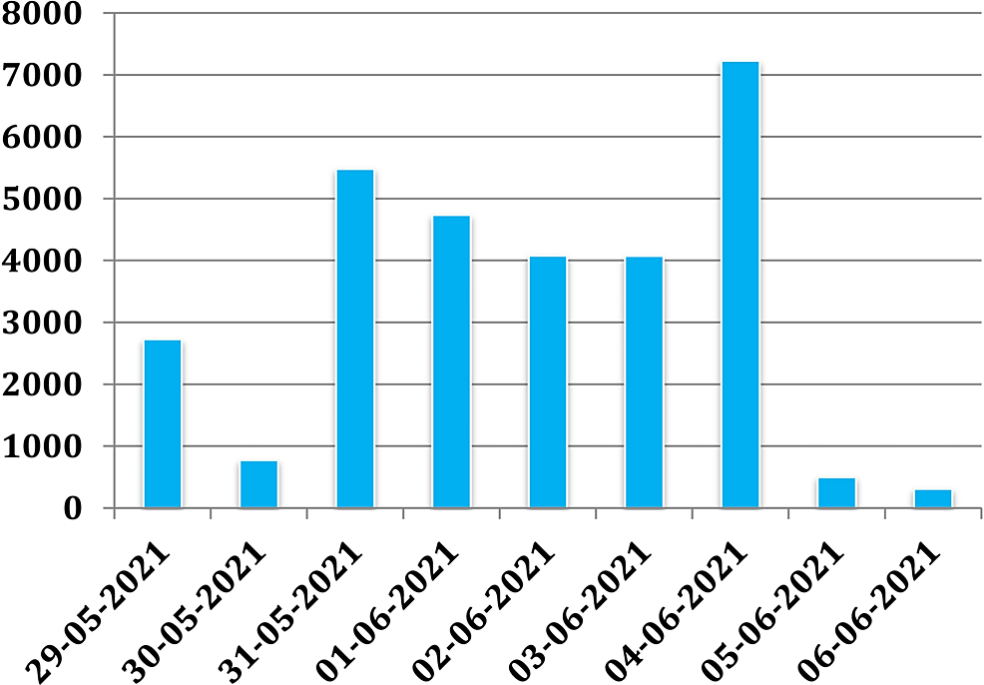
Total Tweet impression of the author who posted the poll (before, during, and after the poll) The polls for the study were posted from 31-05-2021 to 04-06-2021 with the campaign hashtags

We found that when a tweet reaches more users, the number of votes also increases. Similarly, when a higher number of users expand the details or retweet, the number of votes increases. We used the Twitter handle of a user who had 141 followers. Data from Tweet Analytics of the profile showed that the impression had increased (Figure 3) when the polls were posted with the hashtags in the Twitter campaign. Hence, a health-related survey may be coupled with a widely circulated hashtag campaign. This will boost the response to the survey [15].

Although Twitter allows keeping the poll live for seven days, almost all the impressions, engagement, and votes come on the first day of posting the poll. The active users in a campaign may vote as soon as they find the new poll with the followed hashtags. Furthermore, the highest tweet reach may be influenced by the Twitter algorithm [16]. The surveyor can invest a single day to get the result of the poll. However, we still suggest keeping the health-related survey poll for a maximum period to not miss any potential respondents. In this period, the survey poll may be promoted in different channels to increase the response rate [17].

Preference of systems in medicine

For the treatment of common illnesses, users prefer modern medicine, traditional medicine, and self-medication similarly. Although the first choice was traditional medicine, the preferences did not show any statistically significant difference. Preference for self-medication in a common illness may be influenced by lack of time, lack of access to healthcare systems, and financial conditions [18]. However, we did not provide an option of ‘combination of modern medicine and traditional medicine’ for this poll. We intentionally omitted the option just to know the first preference among modern, traditional, and self-medication as a common illness has limited course and a combination is rarely feasible. For the treatment of chronic diseases, a combination of modern medicine and traditional medicine was the preferred system. Chronic diseases take a long course and the patients have the cafeteria choice to choose from different systems of medicine [19]. However, for emergency medical care, modern medicine is preferred over traditional medicine [20]. For the treatment of cancer and treatment of sexual dysfunction or sexually transmitted infections choice of modern medicine and a combination of modern and traditional medicine are equal. The finding of this pilot study would help us to design a large-scale survey to understand which system of medicine people prefer and why they prefer it in the future.

Novelty and limitation of the study

This study reported the feasibility of using Twitter polls for health-related surveys. The results of this study would help public health experts to explore a new avenue for health-related surveys. Although the study was conducted meticulously, it has several limitations. The study was conducted with a particular hashtag campaign. Hence, the poll might have reached a particular group of interested people. This makes the sample of the study to be a non-probability one. Hence, the result of this study may not be extended to the population, and the generalization of the study result is limited. Furthermore, the polls were posted by a single user to test if a single user can conduct a survey successfully. We could reach higher respondents if the polls were posted from multiple users participating in the campaign. During the survey, there was a gradual decrement in participation. However, there were no methods to identify why the participation was decreasing.

## Conclusions

A Twitter poll can be used for a carefully designed health-related survey with short questions and a maximum of four response options. The result of the survey is displayed in percentage with real-time data on the poll page. The result and common analytics are freely available on Twitter. Hence, no paid third-party analytics are required for an anonymous health-related survey. While considering the platform, we should also remember that the poll response rate is highest on the first day of posting the poll. If we consecutively post polls with the same hashtag campaign, there may be a gradual decrease in participation. From the poll result, we found that modern medicine was the first choice for emergency medical care. However, for the treatment of cancer, chronic diseases, sexual dysfunctions, or sexually transmitted diseases, a combination of modern and traditional medicine was preferred. For common illnesses, traditional medicine was the first choice.
